# The effect of bovine milk lactoferrin-loaded exosomes (exoLF) on human MDA-MB-231 breast cancer cell line

**DOI:** 10.1186/s12906-023-04045-1

**Published:** 2023-07-08

**Authors:** Reihaneh Ramezani, Mozhdeh Mohammadian, Elaheh Sadat Hosseini, Mehrak Zare

**Affiliations:** 1 Department of Family Therapy, Women Research Center, Alzahra University, Tehran, Iran; 2 Department of Hematology and Cell Therapy, Faculty of Medical Sciences, Tarbiat Modares University, Tehran, Iran; 3 Department of Genetics, Faculty of Life Sciences, Tehran North Branch, Islamic Azad University, Tehran, Iran; 4 Skin and Stem Cell Research Center, Tehran University of Medical Sciences, Tehran, Iran

**Keywords:** Lactoferrin, Exosome, Target therapy, Drug delivery, Breast cancer, MDA-MB-231

## Abstract

**Background:**

Cancer is still the most challenging disease and is responsible for many deaths worldwide. Considerable research now focuses on targeted therapy in cancer using natural components to improve anti-tumor efficacy and reduce unfavorable effects. Lactoferrin is an iron-binding glycoprotein found in body fluids. Increasing evidence suggests that lactoferrin is a safe agent capable of inducing anti-cancer effects. Therefore, we conducted a study to evaluate the effects of the exosomal form of bovine milk lactoferrin on a human MDA-MB-231 breast cancer cell line.

**Methods:**

The exosomes were isolated from cancer cells by ultracentrifugation and incorporated with bovine milk lactoferrin through the incubation method. The average size of the purified exosome was determined using SEM imaging and DLS analysis. The maximum percentage of lactoferrin-loaded exosomes (exoLF) was achieved by incubating 1 mg/ml of lactoferrin with 30 µg/ml of MDA-MB-231 cells-derived exosomes. Following treatment of MDA-MB-231 cancer cells and normal cells with 1 mg/ml exoLF MTT assay applied to evaluate the cytotoxicity, PI/ annexin V analysis was carried out to illustrate the apoptotic phenotype, and the real-time PCR was performed to assess the pro-apoptotic protein, Bid, and anti-apoptotic protein, Bcl-2.

**Results:**

The average size of the purified exosome was about 100 nm. The maximum lactoferrin loading efficiency of exoLF was 29.72%. MTT assay showed that although the 1 mg/ml exoLF treatment of MDA-MB-231 cancer cells induced 50% cell growth inhibition, normal mesenchymal stem cells remained viable. PI/ annexin V analysis revealed that 34% of cancer cells had late apoptotic phenotype after treatment. The real-time PCR showed an elevated expression of pro-apoptotic protein Bid and diminished anti-apoptotic protein Bcl-2 following exoLF treatment.

**Conclusion:**

These results suggested that exoLF could induce selective cytotoxicity against cancer cells compared to normal cells. Incorporating lactoferrin into the exosome seems an effective agent for cancer therapy. However, further studies are required to evaluate anti-tumor efficacy and the underlying mechanism of exoLF in various cancer cell lines and animal models.

## Introduction

Breast cancer is the most common cancer, excluding non-melanoma skin cancer, and the second cause of cancer-related death in women worldwide [1, 2]. Local therapies, like surgery and radiotherapy, and systemic therapies such as chemotherapy, endocrine therapy, and various immunotherapy approaches, are the main therapeutic considerations for breast cancer [3, 4]. Although advances in breast cancer diagnosis and treatment have reduced patient mortality by 38%, it is still incurable in patients who develop metastatic tumors [1]. Therefore, developing innovative approaches that improve the effectiveness of breast cancer therapy without increasing toxicity risk seems necessary.

Recently natural compounds are emerging as fascinating therapeutic agents for cancer therapy [5]. Lactoferrin (Lf), a cationic iron-binding glycoprotein, has been introduced as a promising candidate for cancer treatment [6]. Lf is found in biological fluids, including blood, saliva, tears, mucus, seminal fluid, bronchial secretions, milk, and neutrophils’ secondary granules [7, 8]. The early findings showed a reduced expression of endogenous Lf in different cancer cells in which the restoration of Lf gene expression impaired their growth and metastasis [9, 10]. Interestingly, Lf affects the normal and cancer cells selectively [6]. Both human and bovine Lfs showed a wide range of anti-tumor activity in controlling tumor proliferation, migration, and invasion [11, 12]. This protein positively regulates normal cell proliferation while inhibiting the growth in cancer cell lines by arresting G1 to S phase transition [13]. Lf has also been found to induce apoptosis by up-regulating Fas, caspase 3, and pro-apoptotic proteins, Bid and Bax, in addition to decreasing anti-apoptotic protein Bcl-2 [14–16]. Moreover, Lf modulates the anti-tumor immune response in the tumor microenvironment [17]. It is reported that Lf significantly prompted NK cells-mediated cytotoxicity against breast and colon cancer cell lines [18]. Up-regulation of NF-kβ signaling and down-regulation of IL-8, IL-6, granulocyte–macrophage colony-stimulating factor (GM-CSF), and TNF-α pro-inflammatory cytokines are associated with anti-inflammatory and immunomodulatory effects of Lf [19]. Overexpression of Lf receptors on cancer cells improves the efficient localization of Lf-based agents in the tumor microenvironment [20, 21]. Furthermore, high bioavailability, safety, immuno-compatibility, and well tolerability are Lf’s advantages for cancer therapy [6].

Although drug selection is crucial for cancer treatment, drug delivery is also critical for successful cancer therapy and optimal therapeutic effects [22]. Furthermore, concurrent use of target therapy and the target delivery system can distinguish normal cells from cancerous cells and reduce the adverse effects of cancer therapy on normal cells [23]. Among the wide range of drug delivery platforms, nanoparticles, especially exosome, is a promising tool for optimal cancer therapy. Since these extracellular vesicles are essential in the body’s cell–cell communications, they can be considered for delivering therapeutic drug cargo [22]. Exosomes are biocompatible substances representing low immunogenicity, high biodistribution, and good permeability [24].

In the present study, we utilize both lactoferrin and exosome capacities to develop an anti-cancer agent. In this regard, we first design an exosome-based drug by delivering Lf into the exosome. Then, we assayed the anti-tumor effects of Lf- contained exosome on the MDA-MB 231breast cancer cell line.

## Methods

### Cell line

The human MDA-MB-231 breast cancer cell line was purchased from the Stem Cell Research Center, STRC (Tehran, Iran) and human adipose-derived mesenchymal stem cells (MSC) were obtained from Skin and Stem Cell Research Center (SSRC). MDA-MB-231 and MSC cells were cultured in Dulbecco's Modified Eagle Medium (DMEM) containing L-glutamine (2 mM), penicillin (100 U/ml) and streptomycin (100 μg/ml) supplemented with 10% fetal bovine serum (FBS) and incubated at 37 °C in 5% CO2. The MDA-MB-231 cells supernatant were collected when 90% confluent and stored at -20 °C until exosome extraction.

### Exosome isolation and characterization

The exosome was isolated from MDA-MB-231 cells supernatant using the ultracentrifugation (UC) method. In brief, 250 ml supernatant was collected from MDA-MB-231 cells. First, dead cells and cell debris were removed from the supernatant by centrifuging at 1000 × g for 10 min, then 9000 × g for 30 min. Next, the supernatant was centrifuged at 100,000 × g for 1 h at 4 °C. Pellet from the culture supernatant was suspended in 200 ml phosphate buffer saline (PBS), passed through a 0.22 µm filter, and the purified Exosomes were stored at -20 °C. The exosome characterization was assessed by scanning electron microscopy (SEM) and dynamic light scattering (DLS) methods. The exosome morphology was assessed by SEM (AIS2300, Seron Technologies Inc., Korea). One drop of exosome solution was placed on a glass microscope slide, dried at room temperature, and coated with a gold layer. The size distribution of exosomes was analyzed by the DLS method. Briefly, 5μL of purified exosomes was diluted in 500μL PBS, and particle size was measured by the Zetasizer Nano ZS (Malvern Instruments, UK). The concentration of exosomes was determined using Bradford's assay is an indirect quantification approach.

### Loading exosomes with lactoferrin

For generating exoLF using the incubation method, 30 µg/ml of purified exosomes were treated with three different bovin milk lactoferrin doses (0.1 mg/ml, 1 mg/ml, 10 mg/ml, and 20 mg/ml). Then, the samples were incubated at room temperature for 1 h, and the loaded exosomes were evaluated using the Bradford protein assay.

### Determination of lactoferrin encapsulated in purified exosome

Samples containing exoLF were ultracentrifuged at 60,000 × g for 2 h to determine the lactoferrin amount loaded in exosomes. Then, the isolated supernatant was evaluated using the Bradford assay for unloaded lactoferrin concentration. The concentration of unloaded lactoferrin was quantified from a standard linear curve derived from known concentrations (0, 10, 20, 40, 60, 80, and 100 mg/ml) of bovine serum albumin. The percentage of lactoferrin loading was calculated as follows:

$$\mathrm{Encapsulated\;lactoferrin}\;(\mathrm{\%})=\frac{\mathrm{primary\;lactoferrin\;concentration }-unloaded\;lactoferrin\;concentration}{\mathrm{primary\;lactoferrin\;concentration}} x 100$$

The primary lactoferrin concentration is the concentration of lactoferrin that was initially mixed with the exosomes.

### Treating the breast cancer cell line with exoLF

MDA-MB-231 cells were seeded at a density of 10^4^ cells/well in a 96-well plate. After overnight plating, cells were treated with different concentration of exoLF including; 20 mg/ml, 10 mg/ml, 1 mg/ml, 0.1 mg/ml for 48 h at 37 °C in 5% CO_2_. The same concentration of exosomes alone was considered control, and the blank sample was DMEM.

### MTT assay

In order to evaluate the lactoferrin effect on normal and tumor cells, the cell viability MTT assay was used after in vitro treatment with exoLF and LF. MDA-MB-231 cell line (tumoral cell) and Mesenchymal Stem Cells (MSCs) (normal cell) were treated with 0.1, 0.5, 1, 2, 5, 10, 20, and 100 mg/ml concentration of LF and exoLF for 24 h. After overnight incubation, 10 µl of MTT solution was added to each well and incubated for 4 h at 37 °C in 5% CO2. Next, the reaction was stopped by adding 100 µl of Dimethyl sulfoxide (DMSO), and the ELISA reader measured the absorbance at 570 nm after 10 min. Cytotoxic effects (IC50) were calculated from curves constructed by plotting cell survival (%) versus drug concentration (mg).

### Quantification of lactoferrin-induced apoptosis by flow cytometry

Annexin V apoptosis detection kit evaluated cell apoptosis in experimental groups. Initially, MDA-MB-231 cells were seeded at a density of 10^5^ cells/well in a 24-well plate for 24 h. Then, cells were treated with 1 mg/ml of LF and exoLF for 24 h at 37 °C in 5% CO2. According to the instructions of BioLegend's FITC Annexin V Apoptosis Detection Kit, cells were stained with Propidium Iodide (PI) and annexin V- FITC dyes after overnight incubation. MDA-MB-231 cells were harvested and washed twice with PBS. Cells were resuspended in 500 µl of 1X Annexin V binding buffer, then 5 µl of annexin V-FITC and 5 µl of PI were added to the cells and incubated at room temperature for 5 min in the dark. Finally, cells were analyzed using a BD FACSCalibur Flow Cytometer (BD Biosciences, San Jose, CA, USA).

### Quantification of apoptosis-related genes expression by RT-PCR

Besides cell apoptosis evaluation by flow cytometry, the expression of apoptotic genes Bcl2 and Bid were assessed using Real-Time PCR. MDA-MB-231 cells were treated with or without exoLF for 24 h at 37 °C in 5% CO2. According to the manufacturer's protocol, total RNA was extracted from cells using an RNeasy Mini Kit (QIAGEN, Germany). Extracted RNA was qualified by determining absorbance ratio at 260 nm and molecular weight analysis by electrophoresis in 1% agarose gel. Then, a QuantiNova Reverse transcription kit (QIAGEN, Germany) was utilized for complementary DNA (cDNA) synthesis from extracted RNA. In brief, 10 µl of purified RNA were incubated in 2 µl QuantiNova gDNA Removal Mix at 45 °C for 2 min and then placed on ice to effectively reduce contaminating genomic DNA (gDNA). Then, 1 µl of QuantiNova Reverse Transcription Enzyme was mixed with 4 µl of QuantiNova Reverse Transcription Mix, added to 15 µl of purified RNA, and Incubate at 25 °C for 3 min and at 45 °C for 10 min. The reaction was inactivated by incubating at 85 °C for 5 min. The quality and quantity of cDNA were evaluated using a spectrophotometric assay and an absorbance measurement at 260 nm. Real-Time PCR amplification was performed as follows: In a total of 10 μl reactive mixture, 0.5 μl forward primer and 0.5 μl reverse primer, 1 μl cDNA, 5 μl SYBER Green master mix, and 3 μl distilled water were mixed. The reaction was run for 35 cycles, each consisting of early denaturation at 95 °C for 2 min, denaturation at 95 °C for 10 s, annealing at 60 °C for 20 s, and extension at 70 °C for 30 s, followed by a final extension prolonged for 10 min at 70 °C.

The RT-PCR primers sets were as follows: forward, 5´-CCT TGC TCC GTG ATG TCT TTC-3´ and reverse, 5´-GTA GGT GCG TAG GTT CTG GT-3´ for Bcl-2, forward, 5´-GAT GTG ATG CCT CTG CGA AG-3´ and reverse, 5´-CAT GCT GAT GTC TCT GGA ATC T-3´ for Bid, and forward, 5´-AAG GTG AAG GTC GGA GTC AAC-3´ and reverse, 5´-GGG GTC ATT GAT GGC AAC AAT A-3´ for GAPDH (Glyceraldehyde-3-Phosphate Dehydrogenase) as an intrinsic control.

### Statistical analysis

The relative expression of target genes was analyzed by REST software. All tests were performed as triplicates. Statistical studies were calculated using the two-way ANOVA method. *P* value < 0.05 was considered as significant.

## Results

### Characterization of MDA-MB-231 cells derived- exosomes

**Fig. 1:**
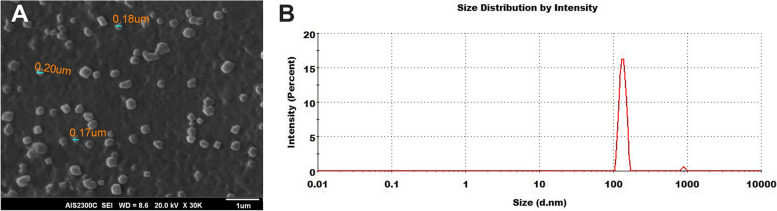
Exosome Characterization. SEM imaging and DLS were utilized to determine isolated exosomes' size distribution and morphology. **A** SEM imaging showed exosomes as round spheroids with an average size between 100 and 200 nm. **B** DLS graph showed an average size of about 100 nm within purified exosomes

Exosome size distribution and morphology were characterized using SEM and DLS. SEM image verified the sphere shape of MDA-MB-derived exosomes and showed the size distributions of purified exosomes from 100 to 200 nm in diameter (Fig. 1A). DLS results also indicated that most exosomes had an average size of 100 nm. The graph is shown schematically in Fig. 1B.

### Efficient loading of lactoferrin in purified exosomes

Exosomes are preferable carriers of drugs, taking advantage of their easy drug loading through exosome-drug co-incubation or sonication and high penetration to target cells through endocytosis and membrane fusion pathways. According to the Bradford assay and its standard curve, the purified exosome concentration was calculated at 230 µg/ml. 30 µg/ml of purified exosomes were incubated with 1, 10, and 20 mg/ml lactoferrin for 24 h. Bradford assay showed the percentage of exoLF, which was 29.72%, 4.2%, and 8.76% at concentrations of 1, 10, and 20 mg/ml of lactoferrin, respectively.

### In vitro* anti-tumor efficiency of exoLF*

**Fig. 2:**
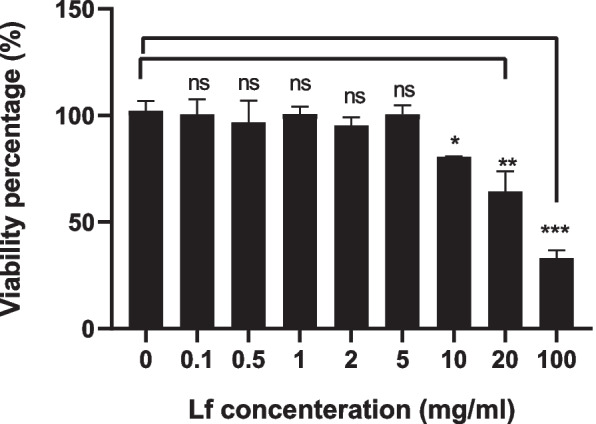
Cytotoxicity effect of free lactoferrin on MDA-MB 231 cell line. The cytotoxicity effects of different concentrations of LF on MDA-MB 231 cell lines were evaluated by MTT assay after 24 h. The lowest cytotoxicity effects of LF on MDA-MB 231 cells occur at 10 mg/ml concentration and reach the maximum at 100 mg/ml concentration. ns: not significant, *: *P* ≤ 0.01, **: *P* ≤ 0.001, ***: *P* ≤ 0.0001

Anti-tumor effects of the lactoferrin delivery via exosomes were evaluated on both MDA-MB 231 cell lines and MSCs. Hence, we first evaluated the cytotoxicity effects of 0.1, 0.5, 1, 2, 5, 10, 20, and 100 mg/ml of free lactoferrin (LF) on MDA-MB 231 cell lines by MTT assay after 24 h. As shown in Fig. 2, the average viability rates of the MDA-MB 231 cells after overnight treatments with 0.1, 0.5, 1, 2, and 5 mg/ml of LF were between 90 and 100%. The viability rate of cells decreased to 80% and 63% at 10 mg/ml and 20 mg/ml, respectively. We observed the minimum cell viability (38%) at 100 mg/ml concentrations.

**Fig. 3:**
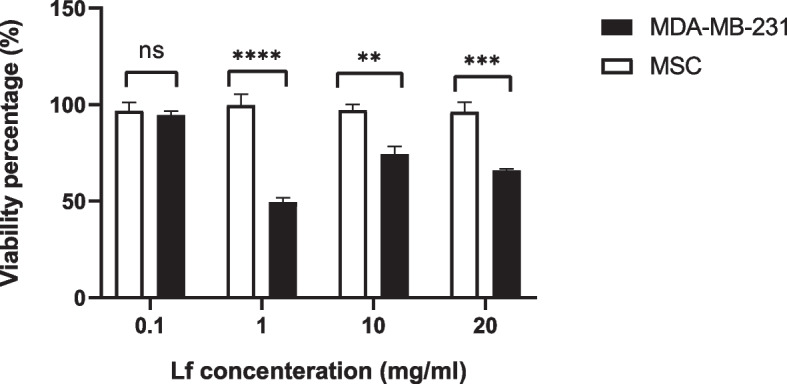
Cytotoxicity effect of lactoferrin-loaded exosomes (exoLF). By evaluating the growth inhibition potency of exoLF on tumoral cells (MDA-MB 231 cell lines) and normal cells (MSCs), we detected 50% cell growth inhibition (IC50) on MDA-MB 231 cell lines at 1 mg/ml of exoLF concentration, compared to other concentrations. None of the 0.1, 1, 10, and 20 mg/ml exoLF could induce a cytotoxicity effect on MSCs. ns: not significant, **: *P* ≤ 0.001, ***: *P* ≤ 0.0001

**Fig. 4:**
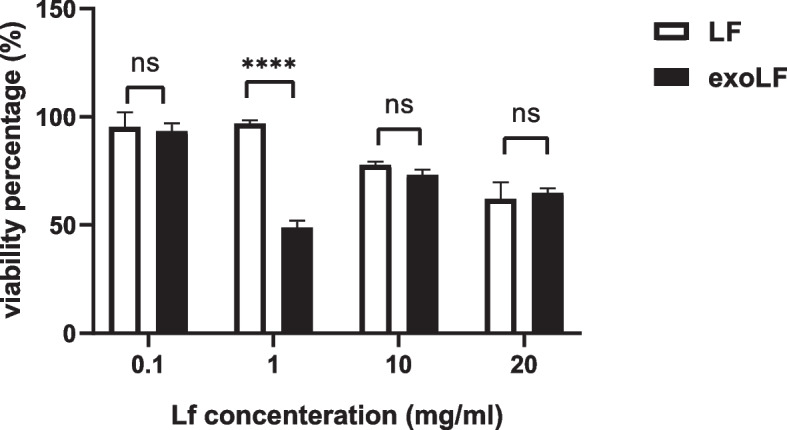
Comparison of the cytotoxic effect of free lactoferrin (LF) and lactoferrin-loaded exosome (exoLF) on MDA-MB 231 cells. There is a significant difference between the effect of LF and exoLF at the concentration of 1 mg/ml. ns: not significant, ***: *P* ≤ 0.0001

**Fig. 5:**
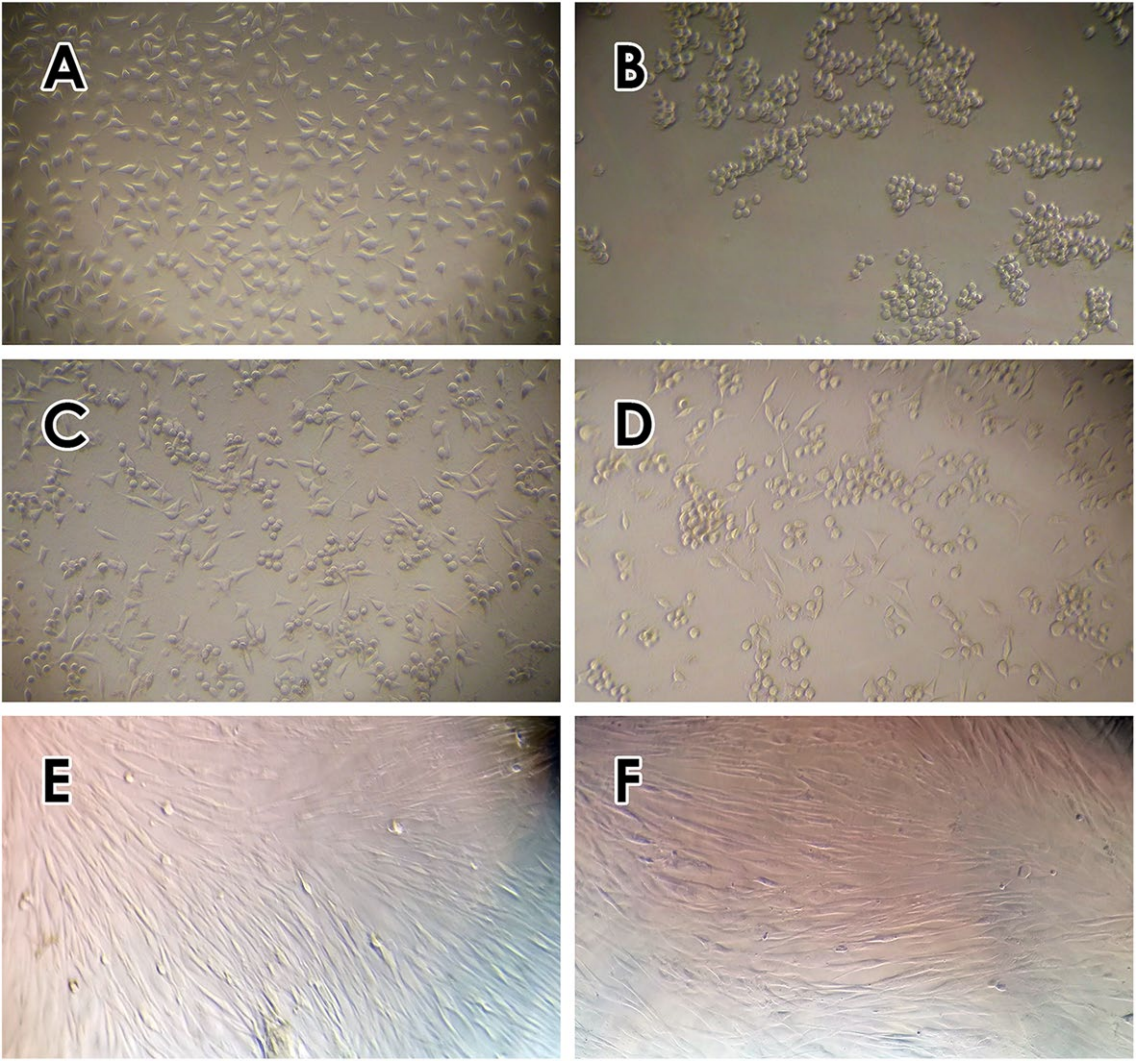
Microscopic examination of exoLF treated MDA-MB 231 cells and MSCs (100 ×). **A** untreated MDA-MB 231 cells, **B**, **C** and **D** treated MDA-MB 231 cells with concentrations **B** 1 mg/ml, **C** 10 mg/ml, and **D** 20 mg/ml exoLF, **E** untreated MSCs and **F** treated MSC cells (1 mg/ml exoLF)

Our results showed that lactoferrin induces its cytotoxicity effects on MDA-MB-231 only at high concenteration (Fig. 2). Therefore, we selected four lactoferrin concentrations (0.1, 1, 10, and 20 mg/ml) to incorporate into the exosome. We observed no growth inhibition on MSCs following treatment with different concentrations of exoLF (Fig. 3). Our results in Figs. 3 and 4 showed that treating MDA-MB 231 cells with exoLF at 1 mg/ml concentration corresponded to 50% cell growth inhibition (IC50).Microscopic imaging of treated and untreated MDA-MB 231 cell lines and MSCs was shown in Fig. 5.

### Lactoferrin-loaded exosomes regulate apoptosis

**Fig. 6:**
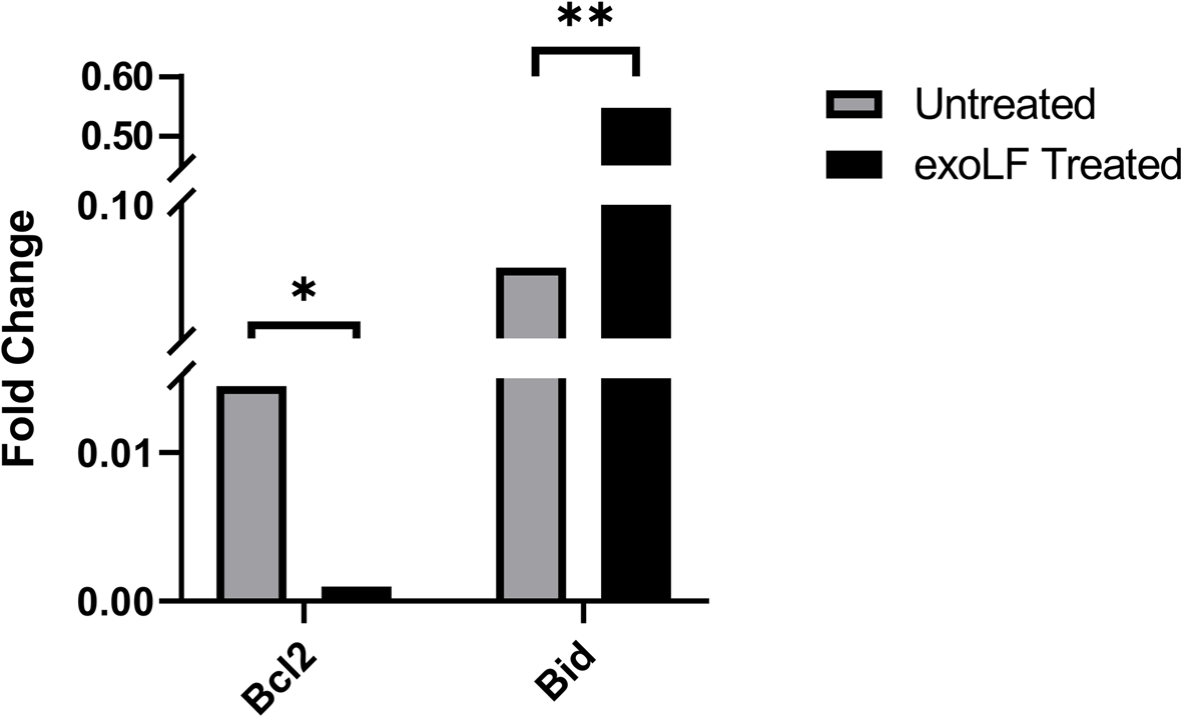
Fold change value of qRT-PCR, a comparison of Bcl-2 and Bid expression between untreated and treated MDA-MB-231 cells. ns: not significant, *: *P* ≤ 0.01, **: *P* ≤ 0.001

To evaluate the functional mechanism of exoLF on tumor cells, we measured the expression level of the Bid pro-apoptotic gene and Bcl-2 anti-apoptotic gene by Real-time PCR. As shown in Table 1, treatment with exoLF led to a reduction of Bcl-2 gene expression by 0.053 fold (*P* v = 0.00) compared with the untreated group. In contrast, the expression of the Bid gene significantly increased by 6.727 fold (*P* v = 0.00) compared to the control (Fig. 5). Figure 6 shows the fold change value of exoLF treated cells and untreated cells.

**Table 1:** Fluctuations in pro-and anti-apoptotic genes expression level

Gene	Type	Reaction Efficiency	Expression	95% C.I	P(H1)	Result
Bcl2	TRG	1.0	0.053	0.023—0.122	0.000	DOWN
Bid	TRG	1.0	6.727	4.000—11.314	0.000	UP
GAPDH	REF	1.0	1.000

**Fig. 7:**
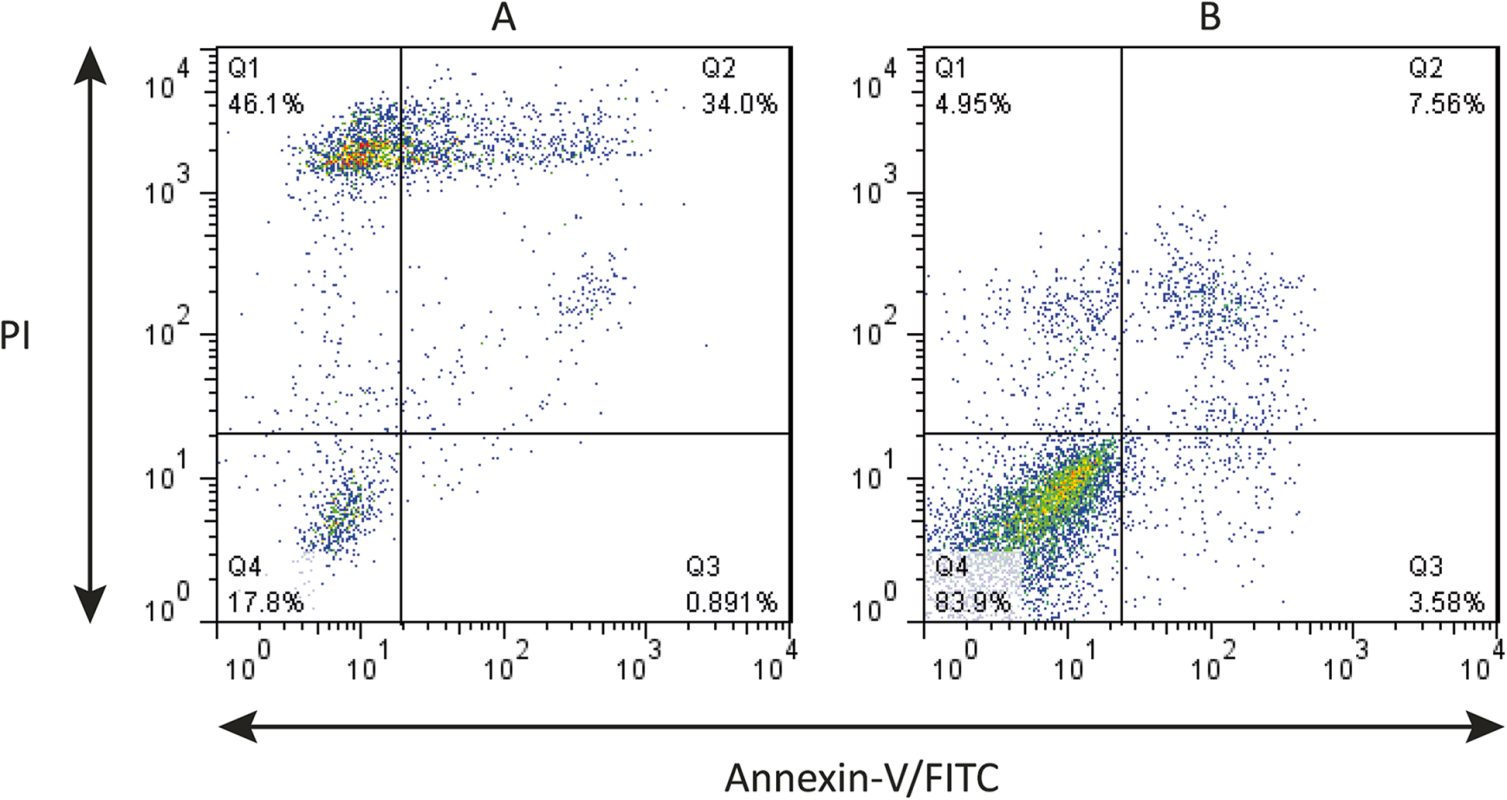
Annexin-V/-FITC/PI flow cytometry analysis of MDA-MB-231 breast cancer cells treated with exoLF. **A** treated cell with exoLF; **B** Untreated cells. The corresponding ExoLF concentration is 1 mg/ml. Annexin V − , PI − : viable cells. Annexin V + , PI − : Early apoptotic cells; Annexin V + , PI + : late apoptotic cells; Annexin V − , PI + : non-viable, necrotic cells

We also verified the cell toxicity of exoLF by flow cytometry analysis. Our results (Fig. 7) showed that at the 1 mg/ml concentration of exoLF, 17.8% of cells were negative for both PI and annexin V (live cells). In contrast, 46% of cells exerted necrotic phenotype (PI + , annexin V -), and 34% had late apoptotic phenotype (PI/ annexin V double positive).

## Discussion

Lactoferrin is a safe, natural food derived from milk protein with potential anti-cancer properties. Interestingly, it could be an appropriate prophylactic anti-cancer because of its determining role in regulating cell growth and the immune system and its anti-inflammatory and anti-oxidant properties [25]. Lactoferrin exerts anti-cancer effects via inducing apoptosis, necrosis, and cell cycle arrest. It also has immunomodulatory activity and inhibits angiogenesis and metastasis. However, its molecular mechanisms are still poorly understood [26]. Bovine lactoferrin has exhibited inhibitory influences on the proliferation of different breast cancer cell lines (MCF-7 cells, T-47D, Hs578T and MDA-MB-231) in a dose-dependent manner [27].

Developing a suitable drug delivery system is one of the critical challenges for achieving optimal target therapy without unfavorable impacts on unspecific cells [28, 29]. Since the exosomes as the extracellular vesicles are essential in the body’s cell–cell communications, they can be considered for delivering therapeutic drug cargo [22]. Exosomes are biocompatible substances representing low immunogenicity, high biodistribution, and good permeability [24].

In the present study, the impacts of lactoferrin loaded exosomes (exoLF) on breast cancer cells have investigated for the first time. First lactoferrin was loaded into MDA-MB 231- derived exosomes and then we compared the antitumor effects of exoLF and free lactoferrin on MDA-MB-231 cancer cells and normal cells. Finally, we evaluated the potential impacts of ExoLF on pro-and anti-apoptotic gene expression.

We used incubation method to load lactoferrin into MDA-MB 231- derived exosomes. Exosomes derived from different cell lines represented protein glyceraldehyde-3-phosphate dehydrogenase (GAPDH), characterized as a lactoferrin receptor [30, 31]. Malhotra and colleagues demonstrated that the GAPDH in exosomes captured lactoferrin and enhanced effective loading of lactoferrin into exosome [32].

Our finding demonstrated that loading lactoferrin into the exosome diminished the optimal concentration of lactoferrin to inhibit tumor growth compared to free lactoferrin. The lower lactoferrin concentration was required for killing MDA-MB-231 cells when it was loaded into the exosome. This result showed that exoLF could transport an adequate amount of therapeutic concentrations of lactoferrin to target cells. Qu et al. indicated that the distribution of dopamine-loaded exosomes was 15-fold higher than free dopamine. Furthermore, treating the mouse model of Parkinson’s disease with dopamine-loaded exosomes was associated with higher therapeutic efficacy than free drugs [33]. Similarly, Aqil and colleagues reported that the anti-tumor efficacy of celastrol against lung cancer cell xenograft had enhanced when celastrol was incorporated into the exosome. Consistent with our results, they claimed that exosomal formulation could improve drug effectiveness and diminish dose-related toxicity [34].

Interestingly, our results revealed that ExoLF exhibited cytotoxicity effects against MDA-MB-231 cells and killed them while the normal cells (MSCs) remained viable. Some chemotherapeutic agents arrest the cell cycle without distinguishing between cancer and normal cells. Novel anti-cancer drugs are necessary to produce specific cytotoxic impacts on cancer cells with minimum or no adverse effects on normal cells [35]. A possible reason for cells’ different responses to lactoferrin could be the difference between the number of expressed lactoferrin receptors on the surface of tumoral and normal cells [36]. Coating nanospheres with lactoferrin was associated with enhanced selective cytotoxicity against MCF-7 and 4T1 breast cancer cells compared to normal fibroblasts [37]. Target selectivity of exosomes against human liver cancer HepaRG cells was shown by Kattawya et al. Their finding indicated that camel milk exosomes lead to apoptosis in cancer HepaRG cells without any adverse effects in normal liver THLE-2 cells [38]. Besides, bovine milk-derived exosomes protected normal intestinal crypt epithelial cells (IEC-6) against oxidative stress agents and promoted normal cell proliferation [39].

We showed that up-expressing Bid and down-regulation of Bcl-2 were involved in MDA-MB-231 cells apoptosis after treatment with exoLF. Similarly, Zhang and colleagues demonstrated the cell cycle arrest due to mitochondrial apoptosis following down-regulation of Bcl-2 and CDC2 in MCF-7 cells by bovine lactoferrin [27]. The elevation of Fas protein expression and raising active forms of caspase 8 and caspase 3 had been associated with bovine lactoferrin-induced apoptosis in the colon carcinoma model [14]. Another study revealed that lactoferrin induced apoptosis in breast cancer cells through inhibiting anti-apoptotic proteins Survivin and Livin. This study also showed that nanoencapsulation of lactoferrin led to selective localization of lactoferrin at the tumor site and improved anti-tumor activity [40]. According to our results, after treatment of MDA-MB-231 cells with exoLF, the little change in Bcl-2 expression was observed but Bid increased significantly. We hypothesize that exoLF has initially induced extrinsic death receptor pathway and then Bid protein plays its role in connecting the death receptor pathway to the mitochondrial pathway. Up-regulation of Fas, caspase 8 and caspase 3 which were proved in Fajita's study, is a confirmation of the action of lactoferrin through the death receptor pathway [14]. In another study, it has been mentioned that full-length lactoferrin (flHLF) after binding to lactoferrin-receptors with Fas-associated death domain (FADD), activates caspase 8, triggering the death receptor pathway [41].

Generally, lactoferrin is a bioavailable, safe, low-cost, and immunocompatible natural compound for cancer therapy. Lactoferrin’s capacity in modulating innate and adaptive immune responses toward improving anti-cancer activity makes lactoferrin a promising candidate for cancer therapy [42]. But due to its potential for degradation, it is necessary to use efficient nanocarriers to protect it from the hydrolytic function of proteases [17] and also to transfer it optimally to the target cells. High drug delivery efficiency, targeting capability, stability, and safety of exosomes are favorable characteristics for using exosomes as drug carriers [43]. In this study, by evaluating the function of exosme as a nanobiocarrier of lactoferrin, we intended to achieve this goal.

## Conclusion

Loading lactoferrin into the exosome seems an effective agent for cancer therapy and exoLF could induce selective cytotoxicity against cancer cells compared to normal cells. However, Future studies will be more informative about the anti-tumor efficacy of exoLF in diverse tumor cell lines. Moreover, establishing suitable experimental animal models to in vivo assess ExoLF anti-tumor effects is required to prove these results.

## Data Availability

The datasets used during the current study are available from the corresponding author on reasonable request.

## Abbreviation

exoLF: Latoferrin loaded exosome
LF: Lactoferrin
MDA-MB-231: Breast cancer cell line
MCF7: Breast cancer cell line
MTT: 3-(4,5-Dimethylthiazol-2-yl)-2,5-diphenyl-2H-tetrazolium bromide
Bax: BCL_2_ Associated X Gene
Bid: BH3 Interacting Domain Death Agonist Gene
Bcl_2_: B-cell lymphoma 2 Gene
DMEM: Dulbecco's Modified Eagle Medium
UC: Ultracentrifugation
FBS: Fetal Bovin Serum
PBS: Phosphate Buffer Saline
SEM: Scanning electron microscope
DLS: Dynamic light scattering
MSC: Mesenchymal Stem Cell
DMSO: Dimethyl sulfoxide
ELISA: Enzyme-linked immunoassay
gDNA: Genomic DNA
GAPDH: Glyceraldehyde-3-phosphate dehydrogenase
IC50: The half maximal inhibitory concentration
PI: Propidium Iodide
IEC-6: Intestinal Epithelial Cell Line 6
THLE-2: Transformed Human Liver Epithelial-2
